# *In vitro* evaluation of the effects of 4-aminopyridine on cytochrome P450 enzymes

**DOI:** 10.3109/21556660.2013.818544

**Published:** 2013-03-26

**Authors:** Anthony Caggiano, Andrew Blight

**Affiliations:** Acorda Therapeutics, Inc., Ardsley, NYUSA

**Keywords:** 4-Aminopyridine, Dalfampridine, Metabolism, Cytochrome P450, Drug-drug interactions

## Abstract

**Background:**

Dalfampridine extended release tablets (dalfampridine-ER, known as prolonged-, modified, or sustained-release fampridine tablets in some countries) are approved for the improvement of walking in patients with multiple sclerosis (MS). Dalfampridine-ER is an extended release formulation of 4-aminopyridine (4-AP). Dalfampridine-ER is incorporated into MS management strategies that may include disease-modifying and symptomatic therapies. Since several symptomatic therapies are partially or fully metabolized by enzymes of the hepatic cytochrome P450 system (CYP450) it is important to evaluate drug–drug interactions through potential effects of dalfampridine-ER on CYP450.

**Methods:**

The ability of 4-AP to inhibit CYP1A2, CYP2A6, CYP2B6, CYP2C8, CYP2C9, CYP2C19, CYP2D6, CYP2E1, and CYP3A4/5 in a direct and time-dependent manner was evaluated using pooled human liver microsomes. 4-AP concentrations were 0.03, 0.1, 0.3, 1, 3, 10, and 30 μM, representing 0.1–100-times the average plasma 4-AP concentration (30 ng/mL; 0.32 μM) at therapeutic dosing; the concentration inhibiting 50% of each enzyme activity (IC_50_) was determined. The ability of 4-AP (0.025, 0.25, 2.5, and 25 μM) to induce the expression of CYP1A2, 2B6, 2C9, 2C19, 2E1, and 3A4/5 enzymes was evaluated using primary cultures of freshly isolated human hepatocytes from non-transplantable livers. The enzyme-inducing effects of 4-AP were compared with the prototypical inducers. Metabolites were assayed using high-performance liquid chromatography-tandem mass spectrometry techniques. All inhibition and induction assays included positive controls.

**Results:**

4-AP did not directly inhibit CYP1A2, CYP2A6, CYP2B6, CYP2C8, CYP2C9, CYP2C19, CYP2D6, or CYP3A4/5, but at a concentration of 30 μM, CYP2E1 was inhibited by 12%, resulting in an estimated IC_50_ value of 125 μM. None of the enzymes demonstrated time-dependent inhibition by 4-AP. There was little or no effect by 4-AP on enzyme induction, with enzyme activities approximately equivalent to vehicle control. A main limitation was the inability to estimate effectiveness of 4-AP relative to prototypical CYP450 inducers.

**Conclusion:**

The likelihood of drug–drug interactions is remote in patients with MS who may be taking dalfampridine-ER concomitantly with medications that are metabolized by CYP450 pathways.

## Background

Dalfampridine extended release tablets (dalfampridine-ER, known as prolonged-, modified, or sustained-release fampridine tablets in some countries), are approved for the improvement of walking in patients with multiple sclerosis (MS)1. This approval was based on two Phase 3 clinical trials that demonstrated a significant improvement in walking speed relative to placebo among patients who responded to treatment2,3.

Dalfampridine-ER is an extended release formulation of dalfampridine or 4-aminopyridine (4-AP), a potassium channel blocker that has been extensively used as a tool for characterizing potassium channels. Its putative mechanism of action in MS is relief of the conduction block in demyelinated axons4, although it may also enhance neurotransmission at synapses5,6. Since this drug is incorporated into MS management strategies that may include a number of other drugs, including disease-modifying therapies (DMTs), it is important to consider its potential for drug–drug interactions.

Many drug–drug interactions are mediated by the hepatic cytochrome P450 system (CYP450). Interferons have been suggested to impact the CYP450 system, thereby potentially altering the pharmacokinetics and pharmacodynamic effects of concomitant medications7. However, the CYP450 system itself is not involved in the metabolism of traditional, injectable DMTs for MS, such as the interferons, glatiramer acetate, and natalizumab, and 4-AP itself is largely excreted unchanged in the urine (discussed below), suggesting low potential risk for drug interactions with concomitant use of dalfampridine-ER. Indeed, in a sub-group analysis of the clinical trials, the tolerability and efficacy of dalfampridine-ER appeared to be similar in patients using and not using these therapies8. Those results suggested that dalfampridine-ER can be effective regardless of DMT use, and is likely to have a safety profile in patients using DMTs that is similar to those not using them. However, in contrast to DMTs, several pharmacologic therapies used for the symptomatic treatment of MS are partially or fully metabolized by enzymes of the CYP450 system, including baclofen and tizanidine for spasticity, tolterodine for neurogenic bladder, dextromethorphan/quinidine for pseudobulbar affect, and a variety of antidepressants that may be used for depression or neuropathic pain. In addition to these treatments related to MS, there may be a variety of other drugs prescribed for concurrent conditions in individual patients.

The pharmacokinetics of 4-AP have been evaluated in several studies9–13, which have included the demonstration that it is rapidly and completely excreted by the urinary route, predominantly as an unchanged compound11. Excretion of unchanged drug suggests that 4-AP does not undergo extensive hepatic metabolism and is therefore not an important substrate of the CYP450 system. However, drug–drug interactions might nevertheless be possible if 4-AP were to inhibit or induce CYP450 enzymes involved in the metabolism of other drugs. Therefore, the purpose of this *in vitro* study was to determine whether 4-AP has an effect, either by induction or inhibition, on components of the CYP450 system that could potentially result in drug interactions. The study was performed in accordance with US Food and Drug Administration (FDA) guidance for performing drug interaction studies14, and includes evaluation of the clinically relevant enzymes CYP1A2, 2A6, 2B6, 2C8, 2C9, 2C19, 2D6, 2E1, and 3A4/5.

## Methods

### Chemicals and reagents

The 4-AP was produced by Regis Technologies (Morton Grove, IL). Stock and working solutions of 4-AP were prepared fresh daily in high-purity water (for inhibition studies) or dimethyl sulfoxide (DMSO).

The following reagents were purchased from Sigma-Aldrich (St. Louis, MO): acetaminophen, 3-amino-1,2,4-triazole, ammonium acetate, bupropion HCl, chlorzoxazone, coumarin, dextromethorphan, dextrorphan, diclofenac, DMSO, furafylline, glucose-6-phosphate, glucose-6-phosphate dehydrogenase, ethylenediaminetetraacetic acid, tetrasodium salt (EDTA), 6-hydroxychlorzoxazone, 7 hydroxycoumarin (umbelliferone), 4′-hydroxydiclofenac, (±)-4′-hydroxymephenytoin, 6*β*-hydroxytestosterone, ketoconazole, magnesium chloride, 8-methoxypsoralen, 4 methylpyrazole, midazolam, modafinil, *α*-naphtho-flavone, *β*-nicotinamide-adenine dinucleotide phosphate (NADP), nicotine, orphenadrine, paroxetine, phenacetin, phencyclidine, quercetin, quinidine, sucrose, sulfaphenazole, testosterone, ticlopidine, Trizma® base and troleandomycin.

Matrigel™ and ITS+ were purchased from BD Biosciences (Bedford, MA). Formic acid was purchased from EMD Chemicals (Gibbstown, NJ). Acetone, acetonitrile, ethanol, hydrochloric acid, methanol, perchloric acid, potassium chloride, potassium hydroxide, sodium chloride and sodium hydroxide were purchased from Fisher Scientific (Pittsburgh, PA). Dulbecco’s Modified Eagle’s Medium (DMEM), GlutaMAX-1, insulin, MEM-non-essential amino acids, modified Eagle’s Medium Dr. Chee’s modification (MCM), and penicillin-streptomycin were purchased from Invitrogen (Grand Island, NY). Carbon dioxide was purchased from Helget Gas (Kansas City, MO). PureCol was purchased from Inamed BioMaterials (Fremont, CA). Fetal bovine serum (FBS) was purchased from SAFC Biosciences (Lenexa, KS). Disodium hydrogen phosphate, potassium dihydrogen phosphate, potassium hydrogen phosphate, sodium hydrogen phosphate, and dipotassium hydrogen phosphate were purchased from Mallinckrodt Baker (Phillipsburg, NJ). Loctite 4013 was purchased from the Loctite Corporation (Rocky Hill, CT). BCA (bicinchoninic acid) Protein Assay Kit was purchased from Pierce Chemical Co. (Rockford, IL). Hydroxybupropion was purchased from BD Gentest (Woburn, MA). 1′ Hydroxymidazolam was purchased from Cerilliant (Round Rock, TX). Tienilic acid was purchased from Cypex Ltd. (Dundee, Scotland). Formic acid was purchased from EMD Chemicals (Gibbstown, NJ). Acetonitrile, acetone, methanol, potassium hydroxide, and sodium hydroxide were purchased from Fisher Scientific (Pittsburgh, PA). Dipotassium hydrogen phosphate and potassium dihydrogen phosphate were purchased from Mallinckrodt Baker (Phillipsburg, NJ). Amodiaquine and *N*-desethylamodiaquine were purchased from LGC Standards (Teddington, Middlesex, UK). Montelukast was purchased from Sequoia Research Products (Pangbourne, UK). *S*-Mephenytoin was purchased from Toronto Research Chemicals Inc. (North York, Ontario, Canada). High purity water and gemfibrozil glucuronide were prepared at XenoTech, LLC (Lenexa, KS). 17*β-N,N*-Diethylcarbamoyl-4-methyl-3-oto-4-aza-5*α*-androstane-17*α*-carboxamide (4-MA) was a generous gift from Dr G. H. Rasmusson (Merck Sharp & Dohme, Rahway, NJ).

The deuterated internal standards used were d_4_-acetaminophen, d_5_-*N-*desethylamodiaquine, d_3_-dextrorphan, d_6_-hydroxybupropion, d_2_-6 hydroxychlorzoxazone, d_5_-7-hydroxycoumarin, d_4_-4′-hydroxydiclofenac, d_3_-4′-hydroxymephenytoin, d_3_-1′-hydroxymidazolam, and d_3_-6*β*-hydroxytestosterone. The sources of these standards are not provided due to the proprietary nature of this information.

### *In vitro* CYP450 inhibition

The ability of 4-AP to inhibit the drug-metabolizing enzymes CYP1A2, CYP2A6, CYP2B6, CYP2C8, CYP2C9, CYP2C19, CYP2D6, CYP2E1, and CYP3A4/5 in a direct and time-dependent manner was evaluated, as previously established15,16, using human liver microsomes pooled from 16 samples that were prepared and characterized at XenoTech, LLC (Lenexa, KS). In brief, duplicate incubations were conducted at 37 ± 1°C in 400 μL incubation mixtures containing potassium phosphate buffer (50 mM, pH 7.4), MgCl_2_ (3 mM), EDTA (1 mM, pH 7.4), and P450 marker substrates at concentrations approximately equal to their *K*m (Table 1). For CYP3A4/5 inhibition, two substrates were used (testosterone and midazolam) as recommended by the FDA14. Reactions were initiated by addition of an NADPH-generating system containing NADP (1 mM), glucose-6-phosphate (5 mM), and glucose-6-phosphate dehydrogenase (1 Unit/mL). After 5 min, the reactions were terminated by addition of an equal volume of acetonitrile (v/v) containing an appropriate internal standard (Table 1). Precipitated protein was removed by centrifugation (920 *g*, 10 min, 10°C). Calibration and quality control metabolite standards were prepared in zero time incubations.

**Table 1.** Experimental conditions for measuring inhibition of microsomal CYP450 activity by 4-aminopyridine.

Enzyme	P450 activity	Substrate concentration, μM	Protein concentration, μg/mL	Ionization mode^a^	Mass transition monitored, amu	Internal standard^b^
1A2	Phenacetin *O*-deethylation	40	100	ESI+	152 → 110	d_4_-Acetaminophen
2A6	Coumarin 7-hydroxylation	0.75	12.5	ESI−	161 → 133	d_5_-7-Hydroxycoumarin
2B6	Bupropion hydroxylation	50	100		256 → 238	d_6_-Hydroxybupropion
2C8	Amodiaquine *N*-dealkylation	7.0	100	ESI+	328 → 283	d_5_-*N*-Desethylamodiaquine
2C9	Diclofenac 4′-hydroxylation	6.0	100	ESI−	310 → 266	d_4_-4′-Hydroxydiclofenac
2C19	*S*-Mephenytoin 4′-hydroxylation	40	100	ESI−	233 → 190	d_3_-4′-Hydroxymephenytoin
2D6	Dextromethorphan *O*-demethylation	7.5	100	ESI+	258 → 157	d_3_-Dextrorphan
2E1	Chlorzoxazone 6-hydroxylation	30.0	100	ESI−	184 → 120	d_2_-6′-Hydroxychlorzoxazone
3A4/5	Testosterone 6*β*-hydroxylation	100	100	ESI+	305 → 269	d_3_-6*β*-Hydroxytestosterone
3A4/5	Midazolam 1′-hydroxylation	4.0	50	ESI+	342 → 324	d_3_-1′-Hydroxymidazolam

amu, atomic mass units; CYP450, hepatic cytochrome P450 system; ESI, electrospray ionization. ^a^Indicates the type of ionization (i.e., ESI) and the polarity (+ or −). ^b^All internal standards were deuterated.

The concentrations of 4-AP that were evaluated (0.03, 0.1, 0.3, 1, 3, 10, and 30 μM) are ∼0.1–100-times the average plasma 4-AP concentration (30 ng/mL; 0.32 μM) that was measured in a clinical trial of the therapeutic dose of dalfampridine-ER (10 mg administered twice daily)2. Because of the possibility that 4-AP may bind to microsomal protein or lipids, an attempt was made to use standard conditions of 0.1 mg/mL microsomal protein, 5 min incubation time, and 50 mM phosphate buffer concentration, for all reactions with the exceptions of coumarin 7-hydroxylation and midazolam 1′-hydroxylation assays. For these reactions, slightly different protein concentrations were used (Table 1) to allow the rate of reaction to be measured under initial rate conditions.

To evaluate 4-AP as a direct-acting inhibitor, the pooled microsomes were incubated with the marker substrates in the presence and absence of the range of 4-AP concentrations to determine the concentration inhibiting 50% of enzyme activity (IC_50_) value. For time-dependent inhibition, the same concentrations of 4-AP were pre-incubated with the microsomes and an NADPH-generating system for 30 min. After pre-incubation, the marker substrate was added, and the incubation was continued for 5 min to measure residual P450 activity. All reactions were terminated as described above. Known direct-acting and metabolism-dependent inhibitors were included as positive controls (Table 2)14. Samples were analyzed as described in the Analytical methods section.

**Table 2.** Positive controls for IC_50_ determinations for inhibition of microsomal CYP450 enzyme activity.

Enzyme	Positive control (concentration, μM)	
	Direct inhibition	Time-dependent assay
1A2	*α*-Naphthoflavone (0.5)	Furafylline (1.0)
2A6	Nicotine (300)	8-Methoxypsoralen (0.05)
2B6	Orphenadrine (750)	Phencyclidine (30)
2C8	Montelukast (0.5)	Gemfibrozil glucuronide (25)
2C9	Sulfaphenazole (2.0)	Tienilic acid (0.25)
2C19	Modafinil (250)	Ticlopidine (0.75)
2D6	Quinidine (0.5)	Paroxetine (0.3)
2E1	4-Methylpyrazol (15)	3-Amino-1,2,4-triazole (10,000)
3A4/5	Ketoconazole (0.15/0.075^b^)	Troleandomycin (25^a^/7.5^b^)

CYP450, hepatic cytochrome P450 system; IC_50,_ concentration inhibiting 50% of enzyme activity. ^a^Testosterone 6*β*-hydroxylation. ^b^Midazolam 1′-hydroxylation.

### *In vitro* CYP450 induction

Evaluation of 4-AP as an inducer of the expression of CYP1A2, 2B6, 2C9, 2C19, 2E1, and 3A4/5 enzymes was performed using primary cultures of freshly isolated human hepatocytes with a Matrigel overlay. Of note, CYP2D6 was not examined because this enzyme is recognized by the FDA as being non-inducible14. Hepatocytes were obtained from non-transplantable livers from three individual donors according to previously described methods17. Culture and treatment procedures were performed as described by Madan *et al*.18. The viability of each preparation was analyzed by trypan blue exclusion, and cultures were allowed to adapt for 3 days prior to treatment.

Hepatocytes were exposed to test articles for 3 consecutive days. Each test article was evaluated in each liver hepatocyte preparation. The test articles included vehicle controls of 0.1% DMSO, 0.1% saline, 4-aminopyridine at concentrations of 0.025, 0.25, 2.5, and 25 μM, and prototypical inducers of human CYP450 including omeprazole (100 μM; CYP1A2), phenobarbital (750 μM; CYP2B6), isoniazid (100 μM; CYP2E1), and rifampin (10 μM; CYP2C9, CYP2C19, and CYP3A4/5); DMSO was the vehicle for 4-aminopyridine, omeprazole, phenobarbital, and rifampin, and saline was the vehicle for isoniazid. The concentrations of 4-AP are ∼0.08–80-times the average plasma concentration, 30 ng/mL (0.32 μM), that was determined in a clinical trial of the therapeutic dose of 10 mg twice daily2.

All working solutions were prepared fresh daily prior to hepatocyte treatment. Approximately 24 h following the final treatment, cultures were visualized to confirm morphological integrity prior to isolation of microsomes, which was carried out according to the methods described by Madan *et al*.18.

Microsomal protein concentrations were determined using a BCA Protein Assay Kit (Pierce Chemical Company, Rockford, IL) and a Synergy HT Multi-Detection Microplate Reader (BioTek Instruments, Inc., Winooski, VT). Microsomal incubations were conducted in duplicate at 37 ± 1°C in 200 μL volumes of incubation buffer (pH 7.4) consisting of high purity water, potassium phosphate (50 mM), MgCl_2_ (3 mM), EDTA (1 mM) and the marker substrates, as shown in Table 3. The testosterone incubations also contained 17*β-N,N* diethylcarbamoyl-4-methyl-3-oto-4-aza-5*α*-androstane-17*β*-carboxamide (1 mM) in acetone (0.1% v/v) to inhibit steroid 5*α* reductase19. These preparations were loaded onto a Tecan Liquid Handling System (Tecan, Research Triangle Park, NC), and reactions were initiated by addition of an NADPH-generating system containing NADP (1 mM), glucose-6-phosphate (5 mM), and glucose-6-phosphate dehydrogenase (1 Unit/mL). Incubation times were either 10 min (CYP2C9, 2E1, and 3A4/5) or 30 min (CYP1A2, 2B6, and 2C19), and reactions were terminated by addition of acetonitrile containing the appropriate internal standards (Table 3). Precipitated protein was removed by centrifugation at 920 *g* (10 min, 10°C), and supernatant fractions were analyzed as described in the Analytical methods section.

**Table 3.** Experimental conditions for measuring induction of microsomal CYP450 enzyme activity by 4-aminopyridine.

Enzyme	P450 activity	Substrate concentration, μM	Protein concentration, μg/mL	Ionization mode^a^	Mass transition monitored, amu	Internal standard^b^
1A2	Phenacetin *O*-dealkylation	80	8	ESI+	152 → 110	d_4_-Acetaminophen
2B6	Bupropion hydroxylation	500	80	ESI+	256 → 238	d_6_-Hydroxybupropion
2C9	Diclofenac 4′-hydroxylation	100	8	ESI−	310 → 266	d_4_-4′-Hydroxydiclofenac
2C19	*S*-Mephenytoin 4′-hydroxylation	400	20	ESI−	233 → 190	d_3_-4′-Hydroxymephenytoin
2E1	Chlorzoxazone 6-hydroxylation	500	8	ESI−	184 → 120	d_2_-6′-Hydroxychlorzoxazone
3A4/5	Testosterone 6*β*-hydroxylation	250	8	ESI+	305 → 269	d_3_-6*β*-Hydroxytestosterone

amu, atomic mass units; CYP450, hepatic cytochrome P450 system; ESI, electrospray ionization. ^a^Indicates the type of ionization (i.e., ESI) and the polarity (+ or −). ^b^All internal standards were deuterated.

### Analytical methods

High-performance liquid chromatography-tandem mass spectrometry (HPLC/MS/MS) was performed using proprietary validated methods for the determination of each metabolite; authentic, deuterated metabolite standards were used in all assays and zero-time incubations served as blanks. Mass spectrometry equipment was either an ABI Sciex (Applied Biosystems/MDS SCIEX, Foster City, CA) API 4000, API 3000, or API 2000 instrument with Shimadzu HPLC pumps and autosampler systems. The ionization mode and mass transitions that were monitored are shown in Tables 1 and 3 for inhibition and induction, respectively. The instruments were equipped with an electrospray (TurboIonSpray) ionization source (Applied Biosystems, Foster City, CA) and two LC-10ADvp pumps with an SIL-HTa autosampler and a DGU-14 solvent degasser (Shimadzu Scientific Instruments, Columbia, MD). In the inhibition studies, the HPLC columns included a Waters Atlantis C18 (5 -μm particle size, 50 mm × 2.1 mm) (Waters, Milford, MA) for the analysis of phenacetin *O-*deethylation, coumarin 7-hydroxylation, amodiaquine *N-*dealkylation, diclofenac 4′-hydroxylation, *S-*mephenytoin 4′-hydroxylation, dextromethorphan *O-*demethylation, chlorzoxazone 6-hydroxylation, testosterone 6*β*-hydroxylation, and midazolam 1′-hydroxylation; and a Waters Atlantis T3 (3 -μm particle size, 50 mm × 2.1 mm) (Waters) for bupropion hydroxylation. Analyses of the induction samples included use of a 5 -μm, 100 mm × 2.1 mm, C18 Waters Atlantis column (Waters) for the analysis of phenacetin *O*-dealkylation; a 5-μm, 50 mm × 2.1 mm, C18 Waters Atlantis column (Waters) for bupropion hydroxylation; a 3-μm, 50 mm × 2.1 mm, T3 Waters Atlantis column (Waters) for diclofenac 4′-hydroxylation, chlorzoxazone 6-hydroxylation, *S-*mephenytoin 4′-hydroxylation, and testosterone 6*β*-hydroxylation. All columns were preceded by a direct connection guard column with a C8, 4.0 mm × 2.0 mm cartridge (Phenomenex, Torrance, CA) and were maintained at ambient temperature.

Metabolites were quantified by back-calculation of a weighted (1/x), linear, least-squares regression. The regression fit was based on analyte/internal standard peak-area ratios calculated from calibration standard samples, which were prepared from authentic metabolite standards. Peak areas were integrated with Applied Biosystems/MDS SCIEX (Foster City, CA).

### Statistical analyses

For inhibition studies, the IC_50_ data were processed with a validated, custom software program (DI IC50 LCMS Template version 2.0.3) for Microsoft Excel (Office 2000 version 9.0, Microsoft Inc., Redmond, WA). When inhibition of enzyme activity was observed, the data were processed for determination of IC_50_ values by non-linear regression with XLfit3 (Version 3.0.5, ID Business Solutions Ltd., Guildford, Surrey, UK). This software uses the Levenberg-Marquardt algorithm for non-linear regression fitting of the data to the following 4-parameter sigmoidal-logistic IC_50_ equation:

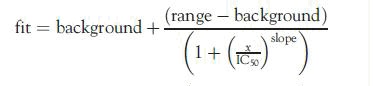



Background was set to 0 with a range up to 100 to express data as a percentage of control. This software has been verified for its ability to calculate IC_50_ values that lie within the concentration range of 4-AP. When less than 50% inhibition is observed, the data are not extrapolated; hence, IC_50_ values are reported as being greater than the highest concentration of 4-AP tested.

For the induction studies, data were processed using the validated, custom software program (EI Interim Data Engine, version 1.2.1) for Microsoft Office Excel 2003 (version 11.0, Microsoft Corporation, Redmond, WA). The individual rates of reaction from replicate samples were averaged, and for those groups with *n* ≥ 3, standard deviations were determined. Statistically significant differences between group means were calculated by equal variance and normality tests to determine if the data were parametrically distributed. For parametrically distributed data sets, with the exception of isoniazid and saline treatment groups, a one-way repeated measures analysis of variance (ANOVA) was carried out to test for significant differences between group means. For isoniazid and saline, a Student’s *t*-test was used to test for differences. For non-parametrically distributed data sets, with the exception of isoniazid and saline treatment groups, a Kruskal-Wallis ANOVA was performed. The ANOVA was followed by a Dunnett’s post-hoc test to identify the group means that were significantly different from the controls. Statistical analyses were performed with Sigma Stat Statistical Analysis System (version 2.03, Systat Software, Inc., Point Richmond, CA). For all analyses, a *p*-value <0.05 was considered to indicate significance. Data were graphed with a validated, custom software program (EI Interim Data Engine, version 1.2.1) for Microsoft Office Excel 2003 (version 11.0, Microsoft Corporation, Redmond, WA). The -fold increase was determined by the enzyme rate for each positive test article divided by the control rate. The enzyme-inducing effects of 4-AP were compared with the prototypical inducers in terms of relative effectiveness, which was calculated by the equation:

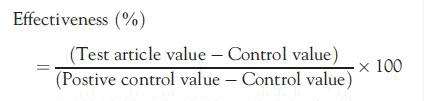



Guidance by the FDA on drug interaction studies suggests that a drug with a change in enzyme activity that results in effectiveness ≥40% relative to the positive control can be considered an enzyme inducer14.

## Results

### *In vitro* CYP450 inhibition

**Figure 1.**
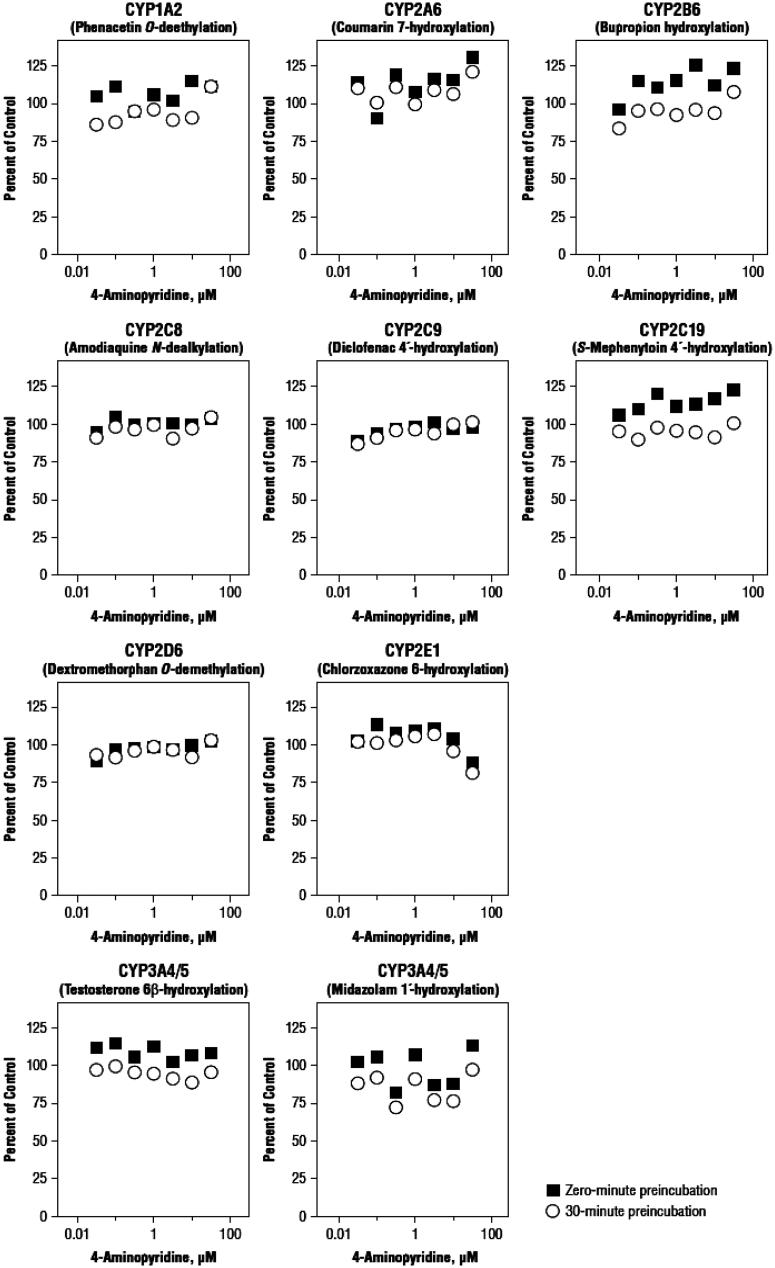
Inhibitory effects of 4-aminopyridine on cytochrome P450 enzymes with and without a 30-min pre-incubation with NADPH-fortified human liver microsomes. Substrate concentrations were approximately equal to their *K*m.

Under the experimental conditions used, there was no direct inhibition of CYP1A2, CYP2A6, CYP2B6, CYP2C8, CYP2C9, CYP2C19, CYP2D6, or CYP3A4/5 at 4-AP concentrations up to 30 μM (Figure 1 and Table 4). However, at a 4-AP concentration of 30 μM, there was ∼12% inhibition of CYP2E1, resulting in an estimated IC_50_ value of 125 μM and a *K*_i_ for the 4-AP-mediated inhibition of CYP2E1 of 62.5 μM; both of which exceed the highest concentration tested.

**Table 4.** *In vitro* effects of 4-aminopyridine on inhibition of human CYP enzymes.

Enzyme	CYP reaction	Direct inhibition; 0-min Pre-incubation		Time-dependent inhibition; 30-min pre-incubation	
		IC_50_, μM	Maximum inhibition at 30 μM, %^a^	IC_50_, μM	Maximum inhibition at 30 μM, %^a^
1A2	Phenacetin *O*-deethylation	>30	ND^b^	>30	ND^b^
2A6	Coumarin 7-hydroxylation	>30	ND^b^	>30	ND^b^
2B6	Bupropion hydroxylation	>30	ND^b^	>30	ND^b^
2C8	Amodiaquine *N*-dealkylation	>30	ND^b^	>30	ND^b^
2C9	Diclofenac 4′-hydroxylation	>30	2.0	>30	ND^b^
2C19	*S*-Mephenytoin 4′-hydroxylation	>30	ND^b^	>30	ND^b^
2D6	Dextromethorphan *O*-demethylation	>30	ND^b^	>30	ND^b^
2E1	Chlorzoxazone 6-hydroxylation	>30	12.0	>30	19.0
3A4/5	Testosterone 6*β*-hydroxylation	>30	ND^b^	>30	4.8
3A4/5	Midazolam 1′-hydroxylation	>30	ND^b^	>30	3.2

CYP, hepatic cytochrome; IC_50,_ concentration inhibiting 50% of enzyme activity. ^a^Maximum inhibition was calculated for the highest concentration of 4-AP based on the following formula: Maximum inhibition (%) = 100% − Percent solvent control. ^b^ND, not determined, since the rates at the highest concentration of 4-AP (30 μM) were greater than the control rates.

When 4-AP was pre-incubated for 30 min in the presence of NADPH-fortified human liver microsomes, there was little or no evidence of time-dependent inhibition for any of the enzymes (Figure 1 and Table 4). Relative to direct inhibition, small increases in inhibition were observed with pre-incubation for CYP1A2, 2A6, 2B6, and C19, although none of these changes was substantial CYP2E1, which had the greatest time-dependent inhibition, and was only inhibited by 19% at 30 μM of 4-AP.

### *In vitro* CYP450 induction

Viability of the hepatocyte preparations was between 83.6% and 91.1%, and these values were considered acceptable for the assays. During and after adaptation to culture, the hepatocytes were judged to be morphologically normal with adequate confluency for treatment. Evaluation of the morphological integrity after final treatment showed that the hepatocytes did not demonstrate any overt signs of toxicity resulting from the test articles.

**Figure 2.**
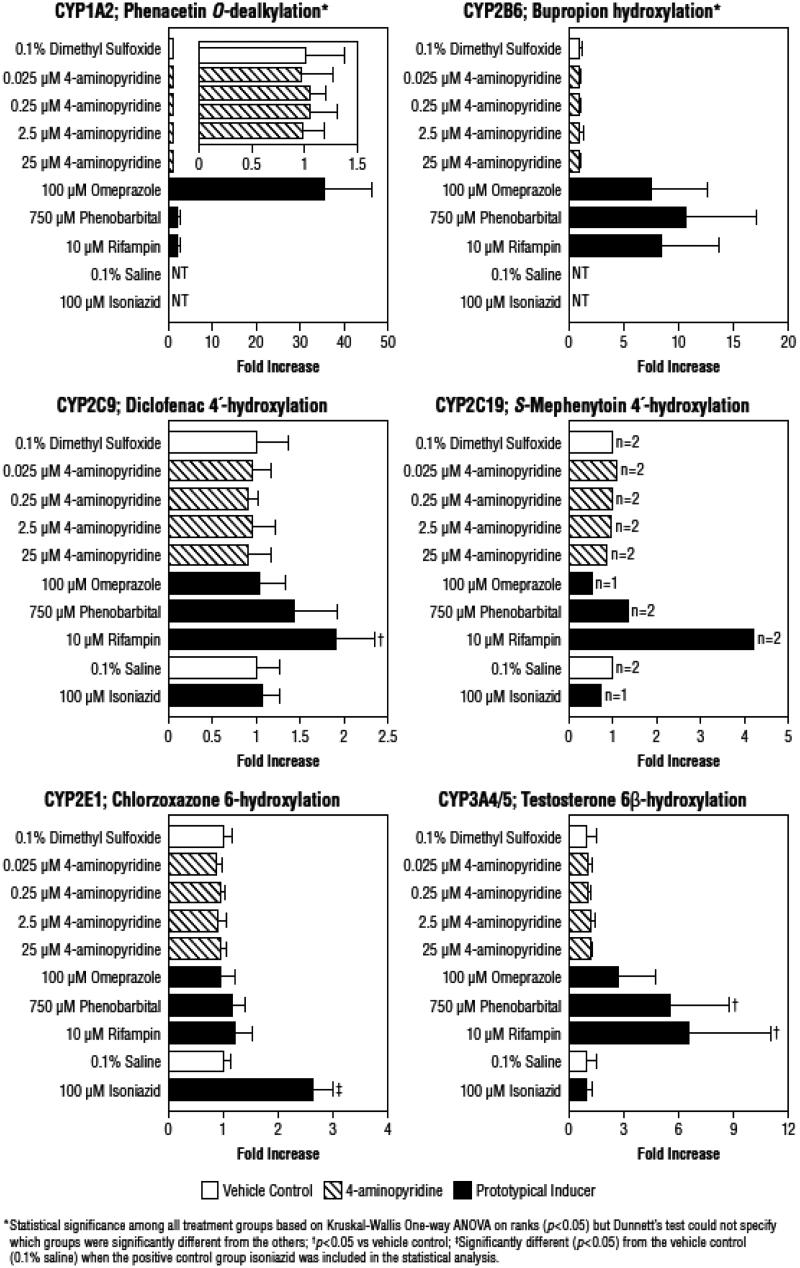
Change in microsomal cytochrome P450 enzyme activity relative to vehicle control after exposure of cultured human hepatocytes to 4-aminopyridine and prototypical inducers relative to vehicle control. Values are presented as -fold increase over vehicle control based on the absolute values shown in Table 3, and are for three determinations (one from each microsomal preparation) unless otherwise indicated.

The mean microsomal CYP enzyme activities after hepatocyte treatment with test articles are summarized in Table 5. As shown in Figure 2, which expresses these activities as the relative increase compared with the vehicle control, hepatocytes responded as expected to treatment with prototypical CYP450 inducers. Treatment with the known inducers resulted in a 35.6-fold increase in activity of CYP1A2 with omeprazole; a 10.7-fold increase in CYP2B6 with phenobarbital; a significant (*p* < 0.05) 2.6-fold increase in CYP2E1 with isoniazid; significant (*p* < 0.05) increases in CYP2C9 (1.9-fold) and CYP3A4/5 (6.6-fold) with rifampin; and a 4.2-fold increase in CYP2C19 with rifampin. For CYP1A2 and CYP2B6, although significance was determined among all the treatment groups as a result of Kruskal-Wallis One-way ANOVA on ranks (*p* < 0.05), Dunnett’s test was unable to specify which individual groups were significantly different.

**Table 5.** Microsomal cytochrome P450 enzyme activity after exposure of cultured human hepatocytes to 4-aminopyridine and prototypical inducers.

Treatment	Concentration	Enzyme activity, mean ± SD^a^, pmol/mg microsomal protein/min					
		1A2	2B6	2C9	2C19	2E1	3A4/5
Dimethyl sulfoxide (vehicle^b^)	0.1% (v/v)	37.0 ± 13.8	38.9 ± 10.4	1350 ± 500	18.2 (*n* = 2)	938 ± 150	2270 ± 1310
4-aminopyridine	0.025 μM	34.9 ± 13.3	37.1 ± 7.7	1250 ± 370	20.0 (*n* = 2)	837 ± 118	2330 ± 1140
4-aminopyridine	0.25 μM	37.7 ± 11.1	36.4 ± 6.3	1190 ± 390	14.3 ± 9.2	880 ± 66	2490 ± 1430
4-aminopyridine	2.5 μM	37.8 ± 14.2	34.9 ± 7.8	1240 ± 310	14.5 ± 10.2	863 ± 227	2560 ± 1260
4-aminopyridine	25 μM	36.1 ± 15.9	35.2 ± 4.0	1170 ± 310	13.2 ± 9.1	897 ± 187	2520 ± 1330
Omeprazole	100 μM	1300 ± 500	280 ± 211	1360 ± 400	8.10 (*n* = 2)	866 ± 117	4440 ± 1370
Phenobarbital	750 μM	74.9 ± 14.6	389 ± 246	1810 ± 280	24.7 (*n* = 2)	1080 ± 170	9960 ± 2260
Rifampin	10 μM	76.4 ± 12.8	309 ± 199	2430 ± 290	61.8 ± 24.3	1120 ± 150	11,100 ± 1500
Saline (vehicle^c^)	0.1% (v/v)	NT^d^	NT^d^	960 ± 253	14.2 (*n* = 2)	268 ± 38	1410 ± 690
Isoniazid	100 μM	NT^d^	NT^d^	991 ± 77	17.9 (*n* = 1)	704 ± 120	1570 ± 960

^a^Values are for three determinations (one from each microsomal preparation) unless otherwise indicated. ^b^Dimethyl sulfoxide was the vehicle for 4-aminopyridine, omeprazole, phenobarbital, and rifampin. ^c^Saline was the vehicle for isoniazid. ^d^NT, not tested due to insufficient protein.

In contrast to the known inducers, 4-AP demonstrated little or no effect on enzymes. Enzyme activities in the presence of 4-AP were approximately equivalent to the DMSO vehicle control, with a very narrow range of increases, 0.89–1.2-fold, across concentrations and enzymes (Figure 2).

Relative effectiveness could not be calculated for 4-AP for CYP2B6 and 2C9, and for three of the four 4-AP concentrations for 2C19 because individual values included negative numbers that precluded determination of an average value (Table 6). However, for CYP1A2 and 3A4/5, as well as 2C19 (2.5 μM concentration only), none of the effectiveness values exceeded the FDA’s cut-off of 40% (Table 6); the highest effectiveness value was 3.29 ± 5.70% for the 2.5 μM concentration for CYP3A4/5. It should also be noted that, since 4-AP was dissolved in DMSO and the prototypical CYP2E1 inducer (isoniazid) was dissolved in saline, the potential of 4-AP to induce CYP2E1 activity could not be evaluated based on effectiveness.

**Table 6.** Relative effectiveness of 4-aminopyridine compared with the prototypical inducers (positive controls expressed as 100% for their respective enzymes). Percent relative effectiveness was calculated as described in Methods.

Treatment	Concentration	Relative effectiveness, mean ± SD				
		1A2	2B6	2C9	2C19	3A4/5
4-aminopyridine	0.025 μM	0.04 ± 0.82	NA^a^	NA^a^	3.10 ± 1.30	0.73 ± 2.38
4-aminopyridine	0.25 μM	0.18 ± 0.50	NA^a^	NA^a^	NA^a^	2.58 ± 2.77
4-aminopyridine	2.5 μM	0.26 ± 0.77	NA^a^	NA^a^	0.44 ± 3.24	3.29 ± 5.70
4-aminopyridine	25 μM	0.05 ± 0.53	NA^a^	NA^a^	NA^a^	2.86 ± 0.41
Omeprazole	100 μM	100	61.9 ± 15.0	NA^a^	NA^a^	25.1 ± 15.9
Phenobarbital	750 μM	3.37 ± 1.45	100	40.6 ± 36.0	11.3 ± 4.1	87.2 ± 8.8
Rifampin	10 μM	3.50 ± 1.58	74.5 ± 6.7	100	100	100

^a^NA, not applicable, individual values included negative numbers that precluded determination of an average value.

## Discussion

Although 4-AP is primarily excreted in the urine as an unchanged drug and is thus not significantly metabolized by CYP enzymes11,20,21, it is important to determine if 4-AP may induce or inhibit CYP activity to further predict if drug–drug interactions may occur with the other drugs regularly used by MS patients. The current analyses confirm that, at therapeutic plasma concentrations of 4-AP, there is little potential of drug–drug interactions through this mechanism. In particular, there were no effects on CYP3A4, which is the most common CYP450 isozyme involved in drug metabolism, and little or no evidence of effects on other evaluated isozymes at 4-AP concentrations that were up to 100-times the mean therapeutic plasma concentration.

While 4-AP did not demonstrate direct inhibitory activity for most enzymes evaluated, there was evidence of low direct inhibition, ∼12%, of CYP2E1 at 30 μM. However, this inhibition was only observed at the highest 4-AP concentration, 30 μM, which is ∼100-times higher than the average plasma 4-AP concentration of 0.32 μM at the therapeutic dose of dalfampridine-ER2, and resulted in an estimated IC_50_ for CYP2E1 of 125 μM. Furthermore, based upon FDA draft guidance14, a ratio of the [I]/*K*_i_ of at least 0.1, where [I] is the concentration of inhibitor exposed to the active site of the enzyme, warrants clinical drug interaction studies. Since [I] would operationally translate to the *C*_max_ (peak plasma concentration), the resulting ratio for 4-AP would be 0.01, suggesting additional drug interaction studies are not warranted based on the estimated *K*_i_ of 62.5 μM for CYP2E1, and a maximum individual *C*_max_ of 67 ng/mL (0.71 μM) that has been reported with therapeutic doses of dalfampridine-ER in a clinical trial2. It should be noted that, although CYP2E1 has been shown to be the predominant enzyme responsible for 3-hydroxylation of 4-AP, the parent compound undergoes very limited metabolic processing22, with clearance primarily as unchanged parent compound in the urine11.

For both CYP2E1 and several other cytochromes, pre-incubation appeared to have a slight impact on inhibition, although there was no substantial increase in CYP2E1 or other cytochrome inhibition relative to the direct assay. However, it is possible that, with longer incubation, a greater inhibitory effect might be observed.

In the induction assays, treatment of three preparations of cultured human hepatocytes with 4-AP at concentrations up to 80-times the therapeutic plasma concentration had little or no effect on CYP1A2, CYP2B6, CYP2C9, CYP2C19, CYP2E1, or CYP3A4/5 activity. FDA guidance suggests that a drug that produces a change ≥40% of the positive control can be considered as an enzyme inducer and warrants *in vivo* evaluation; 4-AP had almost no effect on CYP activity for those values that could be calculated. While 4-AP effectiveness values could not adequately be estimated for CYP2B6, 2C9, 2E1, and 2C19, it may nevertheless be considered unlikely that 4-AP would have a clinically significant impact on the induction of these enzymes since there was no increase in activity with 4-AP relative to the vehicle control, whereas hepatocytes responded as expected to treatment with positive controls.

## Conclusions

This *in vitro* study demonstrated that 4-AP, at concentrations up to 100-fold higher than mean therapeutic plasma concentrations, neither inhibits CYP1A2, 2A6, 2B6, 2C8, 2C9, 2C19, 2D6, 2E1, and 3A4/5 nor induces 1A2, 2B6, 2C9, 2C19, 2E1, and 3A4/5 to a clinically relevant extent. These data suggest that the likelihood of drug–drug interactions is remote in patients with MS who may be taking dalfampridine-ER concomitantly with medications that are metabolized by these pathways.

## Transparency

### Declaration of funding

This study was funded by Acorda Therapeutics, Inc., Ardsley, New York.

**Declaration of financial/other relationship**

AC and AB are employees and stockholders of Acorda Therapeutics, Inc., Ardsley, New York.

## Acknowledgments

The authors wish to thank E. J. Bienen, PhD, of The Curry Rockefeller Group, LLC, Tarrytown, New York, for medical editorial assistance with this report. Editorial support was funded by Acorda Therapeutics, Inc. These studies were conducted at XenoTech, LLC (Lenexa, Kansas).

## References

[1] Ampyra® [dalfampridine] extended release tablets prescribing information. Ardsley, NY: Acorda Therapeutics, Inc., January 2013

[2] Goodman AD Brown TR Krupp L et al Sustained release of oral fampridine in multiple sclerosis: a randomised, double-blind, controlled trial. Lancet 2009;373:732-8 19249634 10.1016/S0140-6736(09)60442-6

[3] Goodman AD Brown TR Edwards KR et al A phase 3 trial of extended release oral dalfampridine in mulitple sclerosis. Ann Neurol 2010;68:494-502 20976768 10.1002/ana.22240

[4] Dunn J Blight A Dalfampridine: a brief review of its mechanism of action and efficacy as a treatment to improve walking in patients with multiple sclerosis. Curr Med Res Opin 2011;27:1415-23 21595605 10.1185/03007995.2011.583229

[5] Lundh H Thesleff S The mode of action of 4-aminopyridine and guanidine on transmitter release from motor nerve terminals. Eur J Pharmacol 1977;42:411-2 15849 10.1016/0014-2999(77)90176-5

[6] Kirsch GE Drewe JA Gating-dependent mechanism of 4-aminopyridine block in two related potassium channels. J Gen Physiol 1993;102:797-816 8301258 10.1085/jgp.102.5.797 PMC2229176

[7] Mahmood I Green MD Drug interaction studies of therapeutic proteins or monoclonal antibodies. J Clin Pharmacol 2007;47:1540-54 17962422 10.1177/0091270007308616

[8] Schapiro R Brown T Goodman A; On Behalf of the MS-F202 M-F, and MS-F204 Study Groups Evaluation of orally administered prolonged-release fampridine tablets for walking improvement in patients with multiple sclerosis treated with disease-modifying therapies. [abstract]. Mult Scler 2010;16(Suppl):S174

[9] Vollmer T Henney HR Pharmacokinetics and tolerability of single escalating doses of fampridine sustained-release tablets in patients with multiple sclerosis: a phase I-II, open-label trial. Clin Ther 2009;31:2206-14 19922891 10.1016/j.clinthera.2009.10.008

[10] Vollmer T Blight AR Henney HR Steady-state pharmacokinetics and tolerability of orally administered fampridine sustained release 10-mg tablets in patients with multiple sclerosis: a 2-week, open-label, follow-up study. Clin Ther 2009;31:2215-23 19922892 10.1016/j.clinthera.2009.10.007

[11] Blight AR Henney HR Pharmacokinetics of 14C-radioactivity after oral intake of a single dose of 14C-labeled fampridine (4-aminopyridine) in healthy volunteers. Clin Ther 2009;31:328-35 19302905 10.1016/j.clinthera.2009.02.004

[12] Smith W Swan S Marbury T et al Single-dose pharmacokinetics of sustained-release fampridine (Fampridine-SR) in healthy volunteers and adults with renal impairment. J Clin Pharmacol 2010;50:151-9 19966074 10.1177/0091270009344857

[13] Henney HR 3rd Blight A Faust B Effect of food on the single-dose pharmacokinetics and tolerability of dalfampridine extended-release tablets in healthy volunteers. Am J Health Syst Pharm 2011;68:2148-54 22058101 10.2146/ajhp110054

[14] United States Food and Drug Administration Guidance for Industry. Drug Interaction Studies — Study design, data analysis, and implications for dosing and labeling. Draft Guidance. US Department of Health and Human Services. Rockville, MD: Center for Drug Evaluation and Research; September 2006

[15] Ogilvie BW Zhang D Li W et al Glucuronidation converts gemfibrozil to a potent, metabolism-dependent inhibitor of CYP2C8: implications for drug-drug interactions. Drug Metab Dispos 2006;34:191-7 16299161 10.1124/dmd.105.007633

[16] Ogilvie BW Usuki E Yerino P et al *In vitro* approaches for studying the inhibition of drug-metabolizing enzymes and identifying the drug-metabolizing enzymes responsible for the metabolism of drugs (reaction phenotyping) with emphasis on cytochrome P450. In: Rodrigues AD, ed. Drug-drug interactions drugs and the pharmaceutical sciences. 2nd edn. New York: Informa Healthcare, 2008 p 231-358

[17] Quistorff B Dich J Grunnet N Preparation of isolated rat liver hepatocytes. In: Pollard JW, Walker JM, eds. Methods in molecular biology. Volume 5. Animal cell culture. Clifton, NJ: Humana Press 1989 p 151-60 10.1385/0-89603-150-0:151 21374123

[18] Madan A Graham RA Carroll KM et al Effects of prototypical microsomal enzyme inducers on cytochrome P450 expression in cultured human hepatocytes. Drug Metab Dispos 2003;31:421-31 12642468 10.1124/dmd.31.4.421

[19] Sonderfan AJ Parkinson A Inhibition of steroid 5 alpha-reductase and its effects on testosterone hydroxylation by rat liver microsomal cytochrome P-450. Arch Biochem Biophys 1988;265:208-18 3415243 10.1016/0003-9861(88)90386-4

[20] Evenhuis J Agoston S Salt PJ et al Pharmacokinetics of 4-aminopyridine in human volunteers. A preliminary study using a new GLC method for its estimation. Br J Anaesth 1981;53:567-70 7248119 10.1093/bja/53.6.567

[21] Uges DR Sohn YJ Greijdanus B et al 4-Aminopyridine kinetics. Clin Pharmacol Ther 1982;31:587-93 7075108 10.1038/clpt.1982.82

[22] Caggiano A Blight A Identification of metabolites of dalfampridine (4-Aminopyridine) in human subjects and reaction phenotyping of relevant cytochrome P450 pathways. J Drug Assessment 2013 10.3109/21556660.2013.833099 PMC4937664 27536445

